# Expression quantitative trait loci associated with performance traits, blood biochemical parameters, and cytokine profile in pigs

**DOI:** 10.3389/fgene.2025.1533424

**Published:** 2025-03-05

**Authors:** Felipe André Oliveira Freitas, Luiz F. Brito, Bárbara Silva-Vignato, Fernanda Nery Ciconello, Vivian Vezzoni de Almeida, Aline Silva Mello Cesar

**Affiliations:** ^1^ Department of Food Science and Technology, Luiz de Queiroz College of Agriculture, University of São Paulo, Piracicaba, Brazil; ^2^ Department of Animal Sciences, Purdue University, West Lafayette, IN, United States; ^3^ Department of Animal Sciences, College of Veterinary Medicine and Animal Science, Federal University of Goiás, Goiânia, Brazil

**Keywords:** gene expression, inflammatory process, eQTL, pig, GWAS, swine, cytokine profile, blood serum indicators

## Abstract

Identifying expression Quantitative Trait Loci (eQTL) and functional candidate variants associated with blood biochemical parameters can contribute to the understanding of genetic mechanisms underlying phenotypic variation in complex traits in pigs. We identified eQTLs through gene expression levels in muscle and liver tissues of Large White pigs. The identified eQTL were then tested for association with biochemical parameters, cytokine profiles, and performance traits of pigs. A total of 41,759 SNPs and 15,093 and 15,516 expression gene levels from muscle and liver tissues, respectively, enabled the identification of 1,199 eQTL. The eQTL identified related the SNP rs345667860 as significantly associated with interleukin-6 and interleukin-18 in liver tissue, while the rs695637860 SNP was associated with aspartate aminotransferase and interleukin-6, and rs337362164 was associated with high-density lipoprotein of the blood serum. In conclusion, the identification of three eQTL significantly associated with aspartate aminotransferase and cytokine levels in both serum and liver tissues suggests a potential role for these variants in modulating immune function and overall health in production pigs. Further research is needed to validate these findings and explore their potential for improving pig health and productivity.

## 1 Introduction

The identification of single nucleotide polymorphism (SNP) located in coding regions of the genome based on mRNA sequencing (RNA-seq) data that are associated with blood biochemical parameters, cytokine profile, and productive traits can contribute to the understanding of genetic mechanisms associated with animal health, welfare, and feed efficiency. These parameters (blood biochemical parameters, cytokine profile, and performance traits) can directly affect economic outcomes. Understanding the genetic factors influencing them allows pig (*Sus scrofa*) breeders and producers to implement targeted breeding and management practices to enhance pig health and productivity (Ye et al., 2018; Ponsuksili et al., 2019; Dall’Olio et al., 2020).

Genome-wide association studies (GWAS) are powerful tools for detecting genomic variants, such as SNPs, associated with complex traits in livestock (Ding et al., 2021; Liu et al., 2021; Wu et al., 2022). In addition to GWAS, information from RNA-seq has been used to explore the transcriptome of specific tissues, offering deeper insights into the effects of genetic variants on traits of interest (Mancuso et al., 2017; Ramayo-Caldas et al., 2019; Li and Ritchie, 2021).

The incorporation of expression quantitative trait loci (eQTL) in GWAS enables the identification of functional candidate variants and a better understanding of their role in genomic and biological processes associated with traits such as disease resistance, metabolic efficiency, and responses to stress (Zhao et al., 2019; Jehl et al., 2021). This integration of GWAS with eQTL analyses has been used for the discovery of candidate variants associated with various traits in pigs, including meat quality indicators such as muscle pH, intramuscular fat (IMF) content, backfit thickness, and lipid profiles (e.g., Chen et al., 2013; Wei et al., 2023). This provides insights into the genetic factors that influence meat quality and triglyceride levels.

Porcine eQTL analyses have also revealed significant associations between genetic variants and the regulation of cytokine expression, which plays a central role in modulating the immune response in humans (Salnikova et al., 2020) and livestock animals (Criado-Mesas et al., 2020; Freitas et al., 2024). According to Salnikova et al. (2020), the mapped SNPs in cytokine genes highlight strong links with inflammatory and immune-mediated diseases. Cytokine-cytokine receptor interactions, such as T cell receptor (TCR) signaling pathways, are involved in the intercellular regulation of the immune system (Kim et al., 2021). Furthermore, genetic variants that regulate immune responses can have direct implications on feed efficiency (Banerjee et al., 2020). This study emphasizes the importance of identifying eQTL that regulates both cytokines and genes associated with metabolism, providing a broader understanding of the genetic basis of health and efficiency in pigs.

The use of indicators that reflect lipid metabolism, immune function, welfare, and health status is essential in pig farming. Blood biochemical parameters, which reflect animal metabolism and health status, are of great significance in pig breeding research and also serve as indirect indicators for productive traits and meat quality in animal production (Song et al., 2022). For example, fatter pigs have higher serum total protein (TP) levels than leaner pigs, and the level of TP is an effective marker for early assessment of fatness in pigs (He et al., 2012; Muñoz et al., 2012). In this context, integrated eQTL and GWAS applied to serum biochemical indicators could allow the exploration of genomic information on economically important traits in pig production.

We hypothesize that eQTLs are associated with performance traits, biochemical blood parameters, and cytokine profiles in Large White pigs. Thus, the primary objectives of this study were to evaluate the association of eQTL with these trait groups in pigs. By utilizing transcriptome sequencing from skeletal muscle and liver tissues of Large White male pigs, we identified cis- and trans-eQTLs and evaluated their association with 34 production, biochemical parameters, and cytokines profile traits in Large White pigs.

## 2 Methods

All experimental procedures involving animals were performed according to the requirements of the Animal Care and Use Committee of the Luiz de Queiroz College of Agriculture (University of São Paulo, Piracicaba, SP, Brazil, protocol: 2018.5.1787.11.6 and number CEUA 2018–28). We also followed ethical principles in animal research, according to the Guide for the Care and Use of Agricultural Animals in Agricultural Research and Teaching (Hill et al., 2020). This study was also conducted in compliance with the ARRIVE guidelines.

### 2.1 Animals, sampling, and mRNA sequencing

A complete description of the experimental animals, phenotypes, sample extraction, and RNA-sequencing of muscle and liver tissues are described in Almeida et al. (2021), Fanalli et al. (2022), and Freitas et al. (2024). Briefly, a total of 72 immunocastrated Large White male pigs (28.44 ± 2.95 kg) were used in a 98-day experimental period. All animals had *ad libitum* access to feed and water throughout the experimental period (98 days). Four days prior to their slaughter, blood samples were taken from all pigs for determination of glucose (GLU; mg/dL), aspartate aminotransferase (AST; U/L), total proteins (TP; g/dL), albumin (ALB; g/dL), globulin (GLOB; g/dL), triglycerides (TG; mg/dL), cholesterol (CHOL; mg/dL), high-density lipoprotein (HDL; mg/dL), low-density lipoprotein (LDL; mg/dL), and very low-density lipoprotein (VLDL; mg/dL).

After 98 days, all pigs were slaughtered (average final body weight of 133.9 ± 9.4 kg), and skeletal muscle (*Longissimus lumborum*) between the 10th and 11th ribs and right lobe of the liver samples were collected within a maximum of 30 min after bleeding. The tissue samples were quickly collected, snap-frozen in liquid nitrogen, and then stored at −80^o^C until further analyses. At slaughter, meat and carcass quality traits were also measured, including slaughter weight (SW; in kg), cold carcass yield as a percentage of the slaughter weight (CCY; %), loin eye area measured by ultrasound (LEA; cm^2^), backfit thickness measured by ultrasound (BFT; cm^2^), intramuscular fat content (IMF, %), and liver fat content (LFC; in %). Furthermore, tissue samples were used to measure the abundance of cytokine levels in skeletal muscle, liver, and blood for Interleukin-10 (IL-10; MFI), interferon-gamma (IFN-γ; MFI), interleukin-1 beta (IL-1β; MFI), interleukin-6 (IL-6; MFI), interleukin-18 (IL-18; MFI), and tumor necrosis factor-alpha (TNF-α; MFI).

### 2.2 RNA extraction, sequencing, and data processing

Skeletal muscle (*Longissimus lumborum*) and right lobe liver tissue samples were collected after slaughter for RNA-seq. Total RNA was extracted from the frozen tissue samples, and the RNA integrity was verified based on RNA integrity number (RIN). Only samples with RIN higher than seven were used. Sequencing adaptors and low-complexity reads were removed using the Trim Galore 0.6.5 software (Krueger et al., 2019), and reads longer than or equal to 70 bases and a *Phred* score threshold greater than 33 were kept for further analyses. After this quality filtering step, alignment and mapping were performed using the current reference pig genome (*Sscrofa* 11.1) (Warr et al., 2020), generating Genomic Variant Call Format (GVCF) (McKenna et al., 2010; Van der Auwera et al., 2013; Franke and Crowgey, 2020) files for each sample from the liver and skeletal muscle tissues.

### 2.3 Variant calling and SNP annotation

The Genome Analysis Toolkit (GATK, v. 4.1.9.0) was used in the GVCF format (Van der Auwera et al., 2013), adopting the HaplotypeCaller algorithm (Van der Auwera et al., 2013) for individually calling the variants for each sample. The output data files with the annotated variants were merged by tissues using the CombineGVCF tool (Van der Auwera et al., 2013; Poplin et al., 2018), and the joint genotyping analysis was performed using the GenotypeGVCF. Subsequently, a VCF file with all samples genotyped for each tissue was obtained and the variants’ annotation and functional consequences were predicted using the Ensembl Variant Effect Predictor tool v. 101 (VEP) (McLaren et al., 2016). The SNP data from muscle, liver, and GGP50K (GeneSeek Genomic Profiler -GGP Porcine 50K, a medium-density SNP chip array with 50,915 SNPs) were merged into a single dataset for subsequent analyses. When the alleles of SNPs between the datasets were different, is the SNP was considered as missing, and both were removed. The variants were filtered based on variant quality scores equal to or greater than 30 (QUAL) and total coverage depth (DP) greater than 10, using BCFtools v. 1.9 (Danecek et al., 2011; 2021; Li, 2011). Moreover, SNP with call rate lower than 95%, minor allele frequency (MAF) lower than 5%, SNPs located in non-autosomal chromosomes, SNPs with extreme departure from the Hardy-Weinberg equilibrium (P < 10^−6^), and non-biallelic markers were removed from the genomic dataset. Finally, linkage disequilibrium (LD) pruning was applied based on a *r* ^2^ threshold of 0.8 within a 100 kb window (Freitas et al., 2024). LD pruning was incorporated into the quality control process to minimize false positives and remove redundant markers. Furthermore, as demonstrated by Freitas et al. (2024), pruning SNPs in linkage disequilibrium can substantially improve the detection of relevant eQTLs in complex traits by reducing confounding effects.

### 2.4 Identification of eQTL

The cis- and trans-eQTL were evaluated using an additive linear model implemented in the Matrix eQTL package (Shabalin, 2012). Principal components (PCs) were fitted as covariates in the models to correct for potential population stratification, along with sire information, dummy categories for treatment effect, and initial body weight as a linear covariate. The expression levels in muscle and liver tissues were tested for association using the combined SNP dataset described above. Gene expression levels were normalized using the average method while preserving rank, a method endorsed by the GTEx consortium (Aguet et al., 2020). Cis-eQTL, defined as local effects, were considered if they were within 1 Mb upstream or downstream of the genes (first and final base pair positions in the gene map). Trans-eQTL were defined as those with a distance greater than 1 Mb from the genes. The model fitted can be defined as:

g=α+γ x+βs+ε,

where g is the gene expression level in transcripts per million (TPM); α is the mean term; γ is the slope coefficient of x , which represents the *k*th covariates, including the top 10 principal components (which explained approximately 28% of the structural population variance), initial body weight in kg as a covariate, sire dummy variables, and categorical effect of treatment dummy variables; β is the slope coefficient of s , which represents the genotype coded as 0 (homozygous for the reference allele), 1 (heterozygous), and 2 (homozygous for the alternative allele), and ε is the residual of the model. We adopted the False Discovery Rate (FDR) correction method for *p*-values (Benjamini and Hochberg, 1995; Huang et al., 2018) with a significance threshold of 0.05.

### 2.5 eQTL association with traits

After the eQTL analyses, significant eQTL were used for association with the biochemical and cytokine profiles in pigs. The traits analyzed included GLU, AST, TP, ALB, GLOB, TG, CHOL, HDL, LDL, VLDL, SW, CCY, LEA, BFT, IMF, LFC, IL-10, IFNg, IL-1β, IL-6, IL-18, and TNF-α in skeletal muscle or liver tissues and blood serum of pigs. The association analysis was conducted using the GCTA software (v.1.94.1) (Yang et al., 2011), employing the mixed linear model (MLMA-LOCO) with the genomic relationship matrix of the animals (GRM). Phenotypic traits were previously adjusted to treatment and block effects. The model used was:

$$y=a+\text{bx}+g^{-}+\varepsilon ,$$

where y represents the trait, a is the observed mean, b is the additive effect (fixed effect) of the SNP or eQTL candidate being tested for association, x is the indicator variable of the SNP genotype coded as 0 (homozygous for the reference allele), 1 (heterozygous), and 2 (homozygous for the alternative allele), $g^{-}$ is the polygenic effect (random effect), representing the cumulative effect of all SNPs, except those located on the chromosome of the candidate SNP (the variance is re-estimated each time when a chromosome is excluded), and ε is the residual effect. The *p*-values resulting from the association analysis were corrected for multiple testing using the FDR method (Benjamini and Hochberg, 1995; Huang et al., 2018) and a significance threshold of 0.05. To illustrate the results, we used the CMplot (Yin et al., 2021) package of the R, to generate the density plot, Q-Q plot, and Manhattan plot in the R environment.

### 2.6 Functional genomic analyses

The functional genomic analyses were performed following Freitas et al. (2024). Briefly, the GALLO R package (Fonseca et al., 2020) was used to perform the QTL annotation and enrichment of the eQTL associated with biochemical parameters and cytokine profiles. The eQTL annotation and enrichment were performed using known QTL data obtained from the PigQTLdb database (Release 53 – *Sscrofa*11.1, 28 Apr 2024), considering a genomic window of up to 500 kb downstream and upstream of the genomic coordinates of the eQTL. The QTL enrichment analyses were performed using a hypergeometric test to reduce the bias of overrepresented traits.

The gene enrichment analysis was performed using the Over-Representative Analysis (ORA) method on the WEB-based Gene Set Analysis Toolkit (Elizarraras et al., 2024; Liao et al., 2019; Elizarraras et al., 2024). The Gene Ontology terms include Biological Processes, Cellular Components (non-redundant), Molecular Functions (non-redundant), and Biological Pathways. The gene list was based on annotations, considering a window of 500 kb up and downstream of significantly associated eQTL genomic coordinates. The gene data annotation of the *Sus scrofa* (Assembly Sscrofa11.1; genome-build-accession GCA_000003025.6; available at: https://ftp.ensembl.org/pub/release-112/gtf/sus_scrofa/) were extracted from the Ensembl platform (Ensembl release 112 - August 2024) (Aken et al., 2016) in the General Transfer Format (“. gtf” format). Finally, multiple protein-protein interaction (PPI) analyses were performed using the STRING 12.0 package (version: 26 July 2023, https://string-db.org/). We explored protein-protein interactions using the gene list with a focus on the *Sus scrofa* species. For that, the same genes annotated around eQTL were used as input. Furthermore, functional genomic annotations were obtained by consulting databases such as UniProt (www.uniprot.org), Ensembl (www.ensembl.org/Sus_scrofa/), and National Center for Biotechnology Information (NCBI, www.ncbi.nlm.nih.gov/).

## 3 Results

### 3.1 SNP data combination and quality filtering

Initially, 84,809 SNPs were identified in liver samples with a genotyping rate of 0.947. Upon merging with the GGP-50k dataset, the number of SNPs increased to 122,325, with a combined genotyping rate of 0.952. Further merging with 75,447 muscle tissue SNPs resulted in 146,344 unique SNPs, and the final genotype rate was 0.926. Quality filtering was then applied to the combined SNP dataset. After removing SNPs with a missing genotype rate greater than 5% (67,861 variants removed), an extreme departure from the Hardy-Weinberg equilibrium test (p-value <10^−6^, 539 variants removed), and MAF below 5% (7,251 variants removed), 70,693 SNPs remained for further analyses. Additional 28,934 SNPs were removed during the LD pruning step and 41,759 SNPs from seventy-two animals remained for further analyses, with a final genotyping rate of 0.989. The detailed process and results of SNP data combination, quality filtering, and LD pruning are provided in Supplementary Tables S1–S3.

**FIGURE 1:**
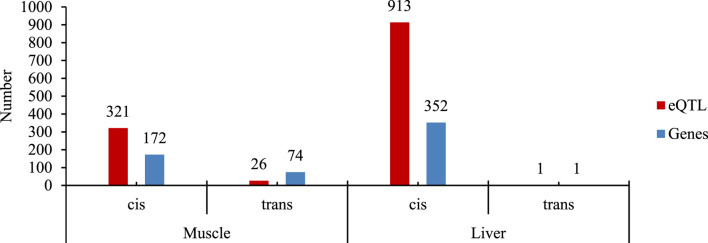
Number of cis- and trans-eQTL and the regulated genes for each tissue evaluated (muscle and liver). The SNP dataset includes genotypes from the GGP-50K plus the RNA-Seq SNP calling of the skeletal muscle and liver tissues after linkage disequilibrium pruning.

The SNPs (n = 41,759) were tested for association with the expression level of 15,093 and 15,516 genes from muscle and liver, respectively. The number of significant eQTL and regulated genes found in muscle and liver tissue are shown in Figure 1, and a summary of eQTL analysis is provided in Supplementary Material 1.

### 3.2 eQTLs associated with biochemical blood parameters and cytokine profiles

**FIGURE 2:**
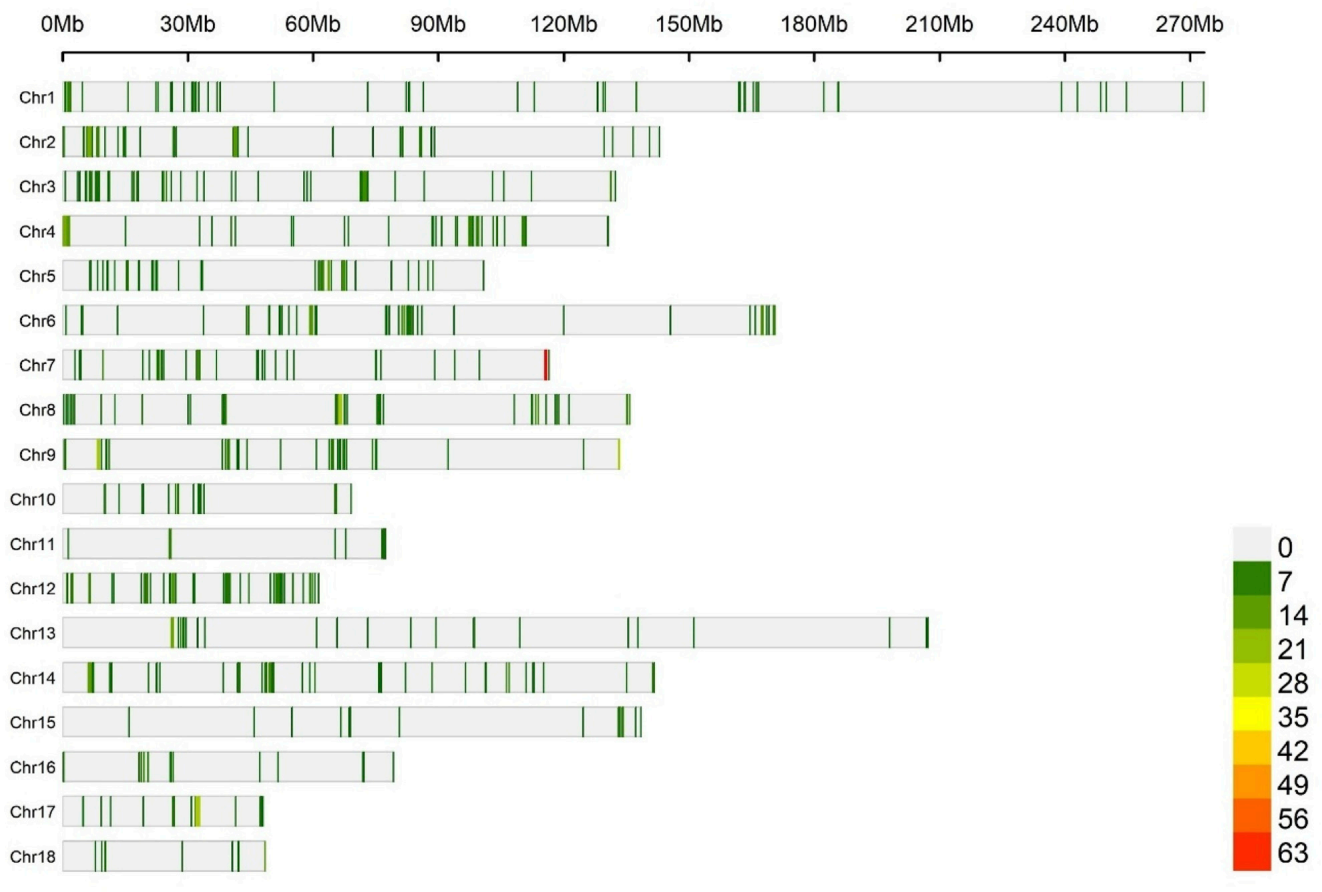
Number of eQTL within 1 Mb window size along the chromosomes, used to test associations with the traits. Each vertical bar represents a genomic window, and the density of eQTLs is indicated by the color scale, ranging from green to red as the eQTL density increases.

Figure 2 shows the number of eQTLs for each 1 Mb window, dispersed across the chromosomes used to test associations with the traits (1,199 eQTLs). The descriptive statistics of the performance traits, blood biochemical parameters, and cytokine profiles of pigs are presented in Supplementary Table S4.

**FIGURE 3:**
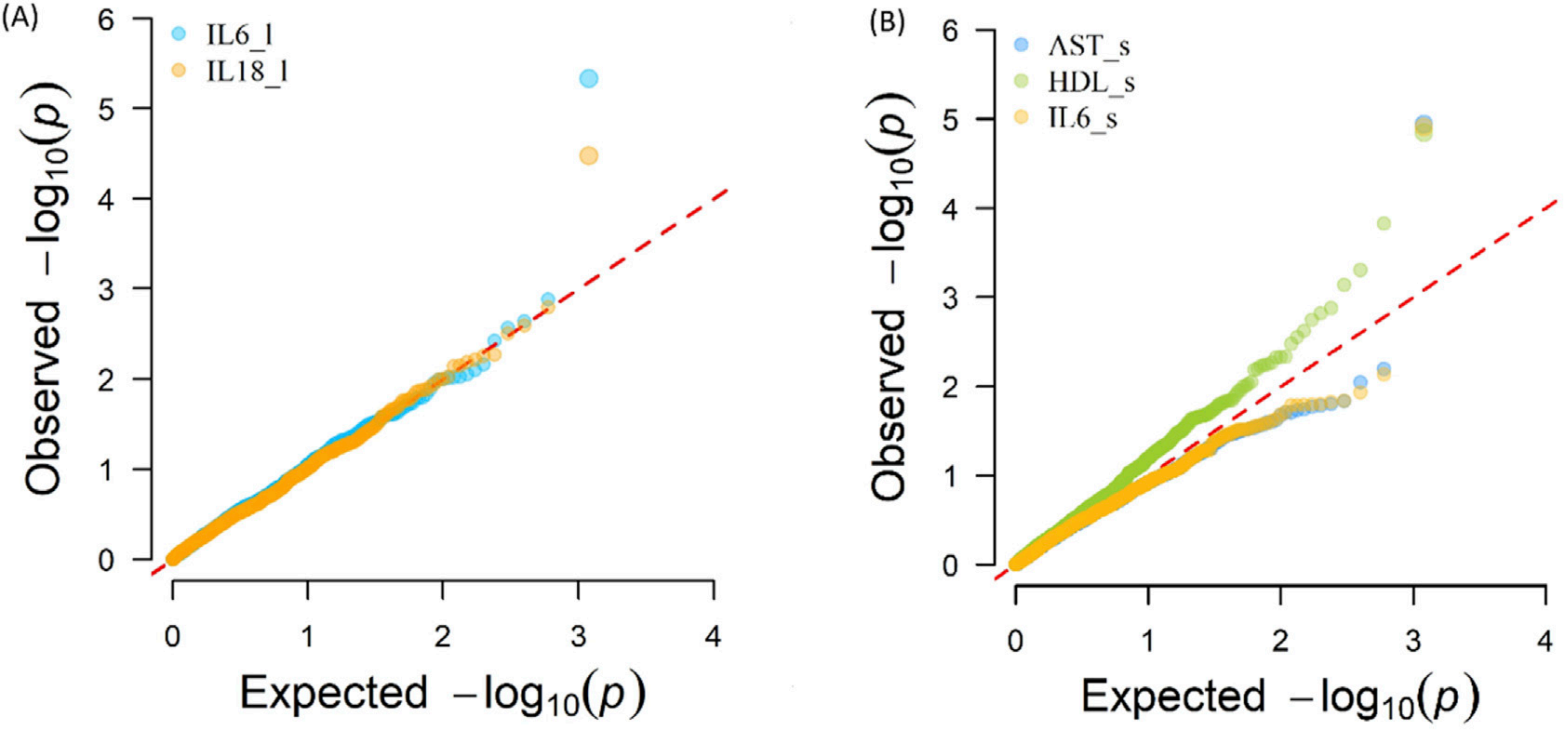
Expected distribution of -log10(p) values *versus* the observed distribution for the phenotypes **(A)** Interleukin-6 (IL6_l, liver tissue, λ = 1.048), and Interleukin-18 (IL18_l, liver tissue, λ = 1.016) **(B)** Aspartate Aminotransferase (AST_s, blood serum, λ = 0.876), High-Density Lipoprotein (HDL_s, blood serum, λ = 1.252), Interleukin-6 (IL6_s, blood serum, λ = 0.878).

The Q-Q plot (Figure 3) shows the expected distribution of -log10(*p*) values *versus* the observed distribution for the phenotypes in blood serum AST, HDL, IL-6, and liver tissue IL-6 and IL-18. A summary of the genomic inflation factors of the p-values resulting from the GWAS analysis with eQTL are presented in Supplementary Table S5.

**FIGURE 4:**
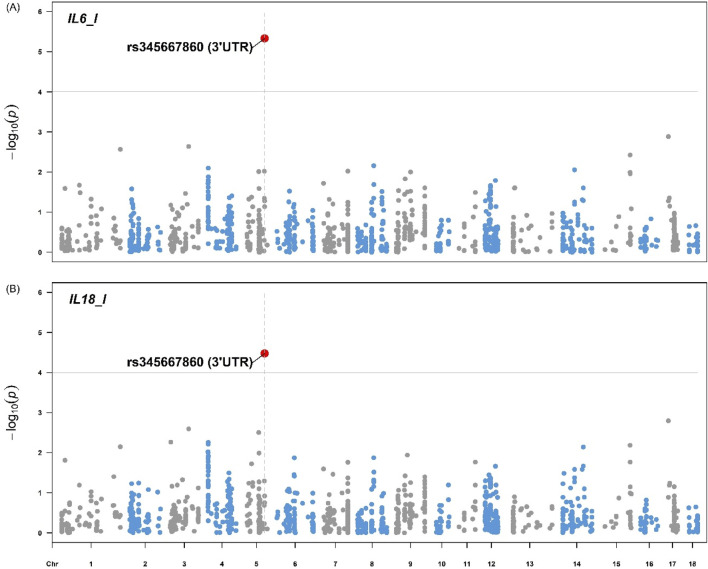
Manhattan plot for the **(A)** Interleukin-6 and **(B)** Interleukin-18 from pigs. The Manhattan plot displays the genomic positions of eQTLs on the x-axis and the -log10(*p*) values on the y-axis for the phenotypes IL-6 and IL-18 in pig liver tissue. The highlighted points represent eQTLs with significant associations based on the FDR <0.05. The variant rs345667860 (3′UTR) is indicated with respective effect predicted using the Variant Effect Predictor (VEP) from Ensembl. IL-6 = Interleukin-6 (MFI), IL-18 = Interleukin-18 (MFI).

**FIGURE 5:**
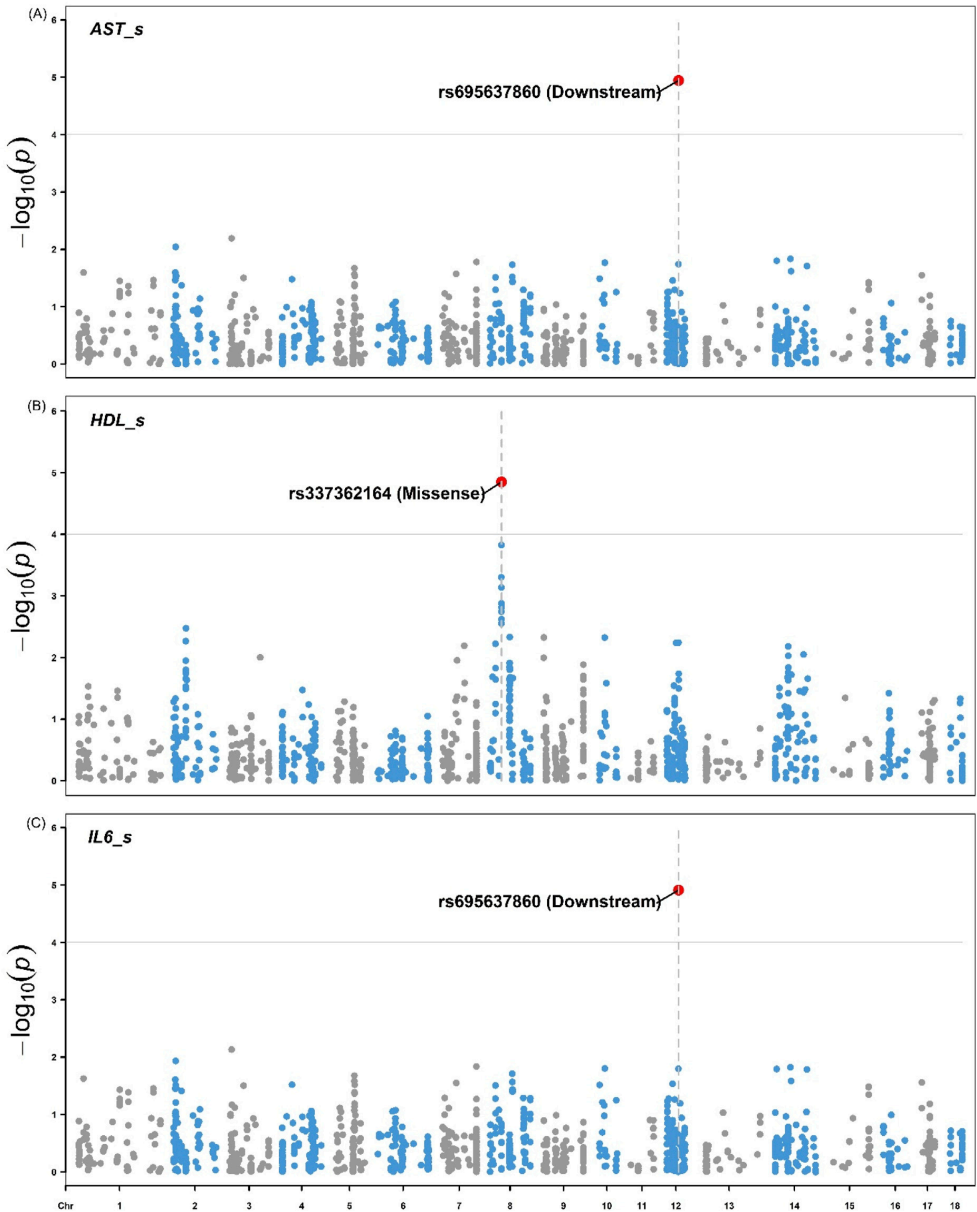
Manhattan plots for the **(A)** aspartate aminotransferase **(B)** High-Density Lipoprotein, and **(C)** Interleukin-6 from pigs. Manhattan shows the distribution of p-values by genomic positions of eQTLs on the x-axis and the -log10(p) values on the y-axis for the phenotype’s aspartate aminotransferase (AST; U/L), high-density lipoprotein (HDL; mg/dL) and interleukin-6 (IL-6; MFI) in pig serum. The highlighted points represent eQTLs with significant associations based on the FDR <0.05 threshold. The variants rs695637860 (Downstream) and rs337362164 (Missense) are indicated with respective effects predicted using the Variant Effect Predictor (VEP) from Ensembl.

Figures 4, 5 illustrate the distribution of eQTLs across the genome for the phenotypes in liver tissue IL-6 and IL-18 (Figure 4), as well as blood serum, AST, HDL, and IL-6 (Figure 5). The highlighted points in the plots represent the significant eQTL. Specific SNPs such as rs345667860 (3′UTR), rs695637860 (Downstream), and rs337362164 (Missense) are indicated, with effects predicted using the Variant Effect Predictor (VEP) from Ensembl.

The Manhattan and QQ plots of all other traits are in Supplementary Material 2 (.zip). Furthermore, the summary statistics table of GWAS, which includes all genomic inflation values is presented in Supplementary Table S5.

### 3.3 Functional genomic analyses

**FIGURE 6:**
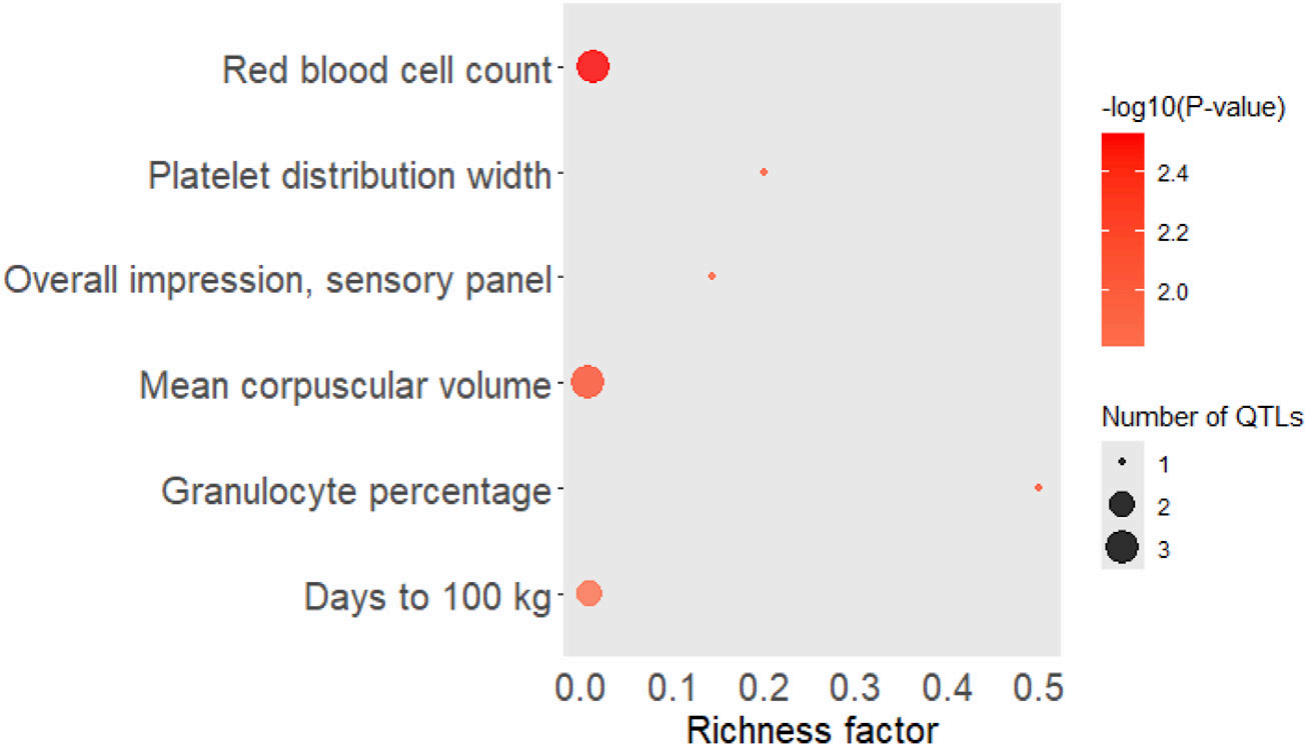
Top significant traits in Production, Health, Meat, and Carcass enrichment analyses around eQTL associated with AST, HDL, IL-6 in pig serum, and IL-18 and IL-6 in pig liver tissue. The area of the bubbles represents the number of observed QTL for that class, while the color represents the p-value scale (the darker the color, the more significant the p-values). Additionally, the X-axis shows the richness factor for each QTL, representing the ratio of the number of QTL and the expected number of that QTL. AST = Aspartate aminotransferase (U/L), HDL = high-density lipoprotein (mg/dL) and IL-6 = interleukin-6 (MFI) in pig serum, IL-6 = Interleukin-6 (MFI), IL-18 = Interleukin-18 (MFI) in pig liver tissue.

The QTL types enriched with the significant eQTLs (FDR <0.05) located at SSC12:39,493,883, 8:39,107,307, and 5:88,678,346 (chromosome:base-pair) was “Health” followed by “Production” and Meat and Carcass.” Top significant traits in Production, Health, Meat, and Carcass enrichment analyses around eQTL associated with AST, HDL, IL-6 in pig serum, and IL-18 and IL-6 in pig liver tissue are shown in Figure 6.

**FIGURE 7:**
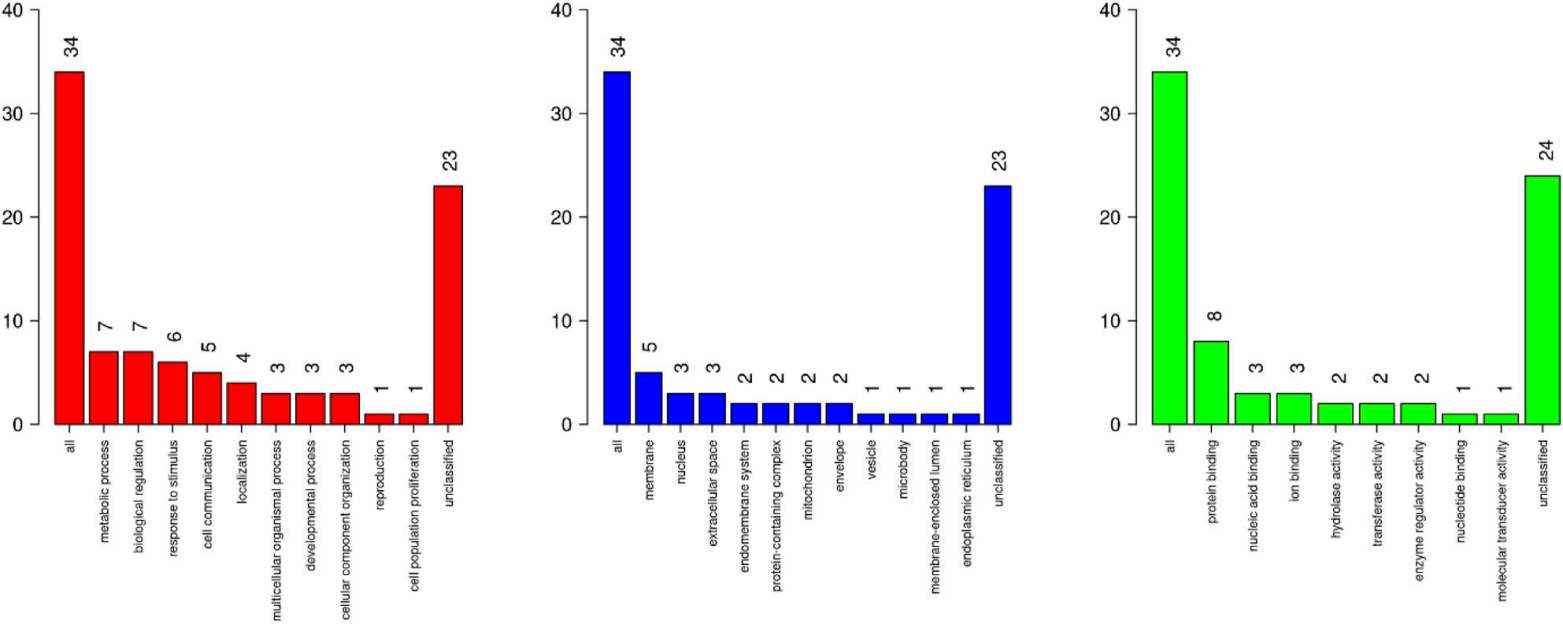
Distribution of Gene Ontology (GO) categories identified in enrichment analysis annotated around eQTLs associated with biochemical parameters and cytokine profiles from pig blood serum and liver tissue. The left chart (red) shows the Biological Process (BP) categories. The middle chart (blue) presents Cellular Component (CC) categories. The right chart (green) illustrates the Molecular Function (MF) category.

The gene enrichment type analysis for the genes around significant eQTL included 34 mapped genes from unique *Entrez gene* IDs. Parameters included a minimum of three gene IDs per category, with an enrichment significance level adjusted to FDR <0.05. The bar charts in Figure 7 show the distribution of these Gene Ontology categories (Biological Processes–BP, Molecular Functions–MF, and Cellular Components–CC).

**FIGURE 8:**
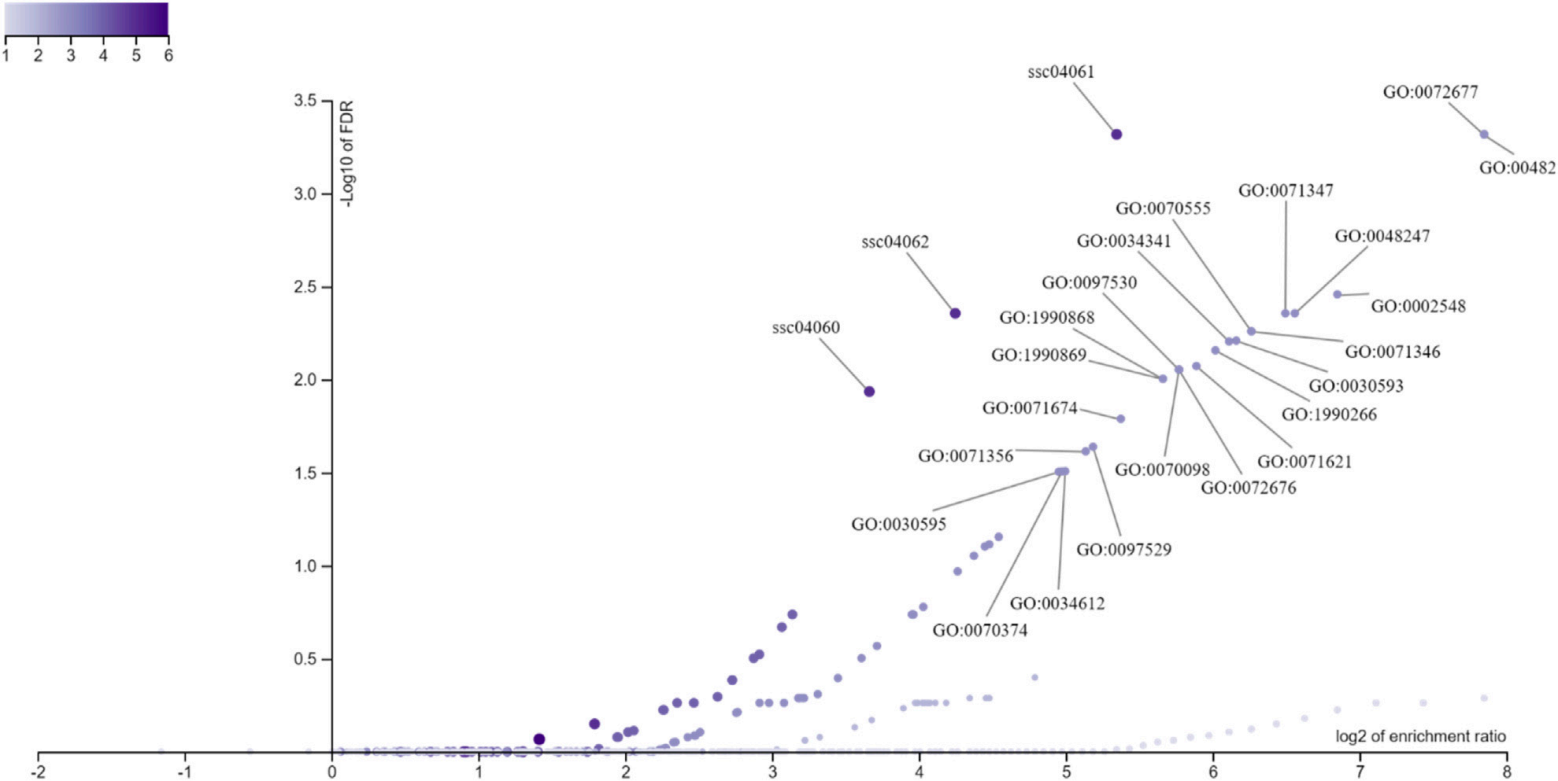
Distribution of Gene Ontology (GO) terms and metabolic pathways terms (MP) based on gene enrichment analysis annotated around eQTLs associated with biochemical parameters from pig blood serum and liver tissue. The x-axis displays the log_2_ of the enrichment ratio, indicating the magnitude of enrichment, while the y-axis shows the -log_10_ of the FDR, representing the statistical significance. Each point on the plot corresponds to a specific GO term or pathway, with points further to the right and higher on the plot indicating terms with both high enrichment and strong significance. The color gradient represents varying levels of enrichment, with darker colors indicating higher enrichment scores.

The significant GO (Gene Ontology) and MP (Molecular Pathway) terms are shown in Figure 8, where the x-axis corresponds to the log_2_ enrichment ratio relative to the -log_10_ FDR on the y-axis for genes annotated around the eQTL associated with biochemical parameters from pig blood and liver tissue.

**FIGURE 9:**
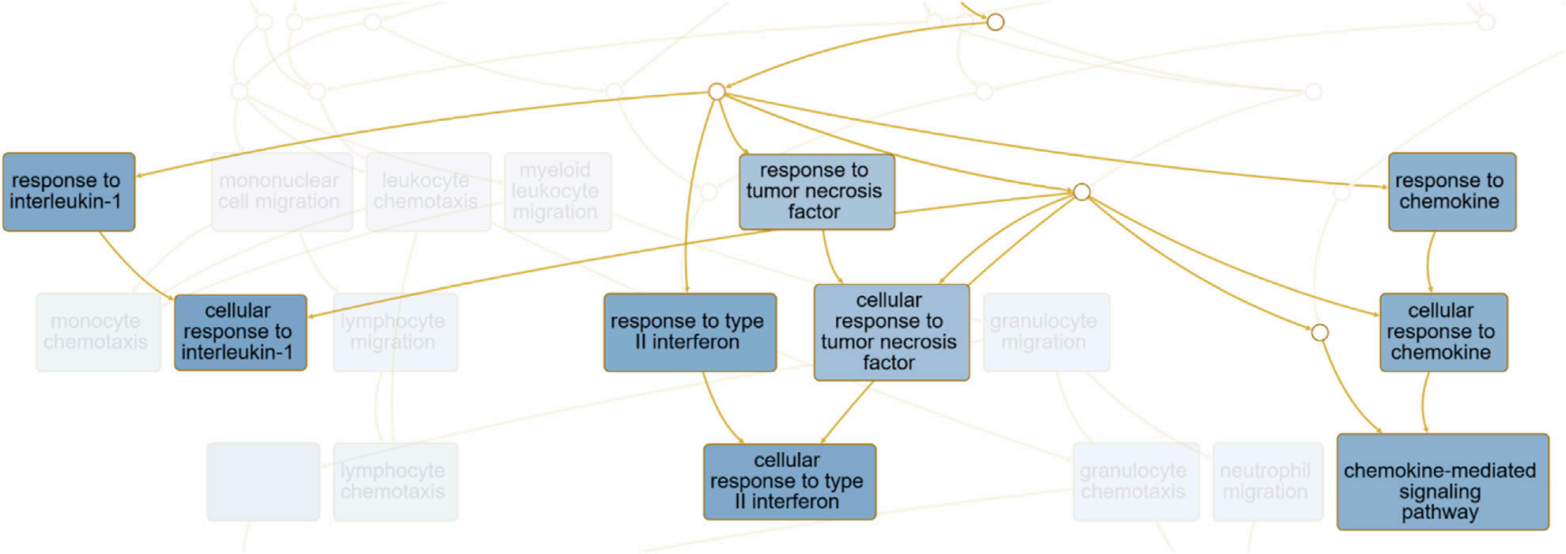
The hierarchical structure of biological processes identified through enrichment analysis for biological processes ontology terms. Highlighted nodes correspond to processes related to “Response to cytokine–GO:0034097”, and the complete directed acyclic graph (DAG) with all GO terms and metabolic pathways is available in Supplementary Figure S1.

For better understanding the enrichment analysis, a directed acyclic graph (DAG, output report for ORA from WebGestalt, the complete results can be accessed on https://2024.webgestalt.org/results/1732400034/) was generated to describe the hierarchical relationships between the enriched biological processes, and the full version can be viewed in Supplementary Figure 1 (.png). We highlighted the nodes corresponding to “Cytokine Response–GO:0034097” in Figure 9.

**FIGURE 10:**
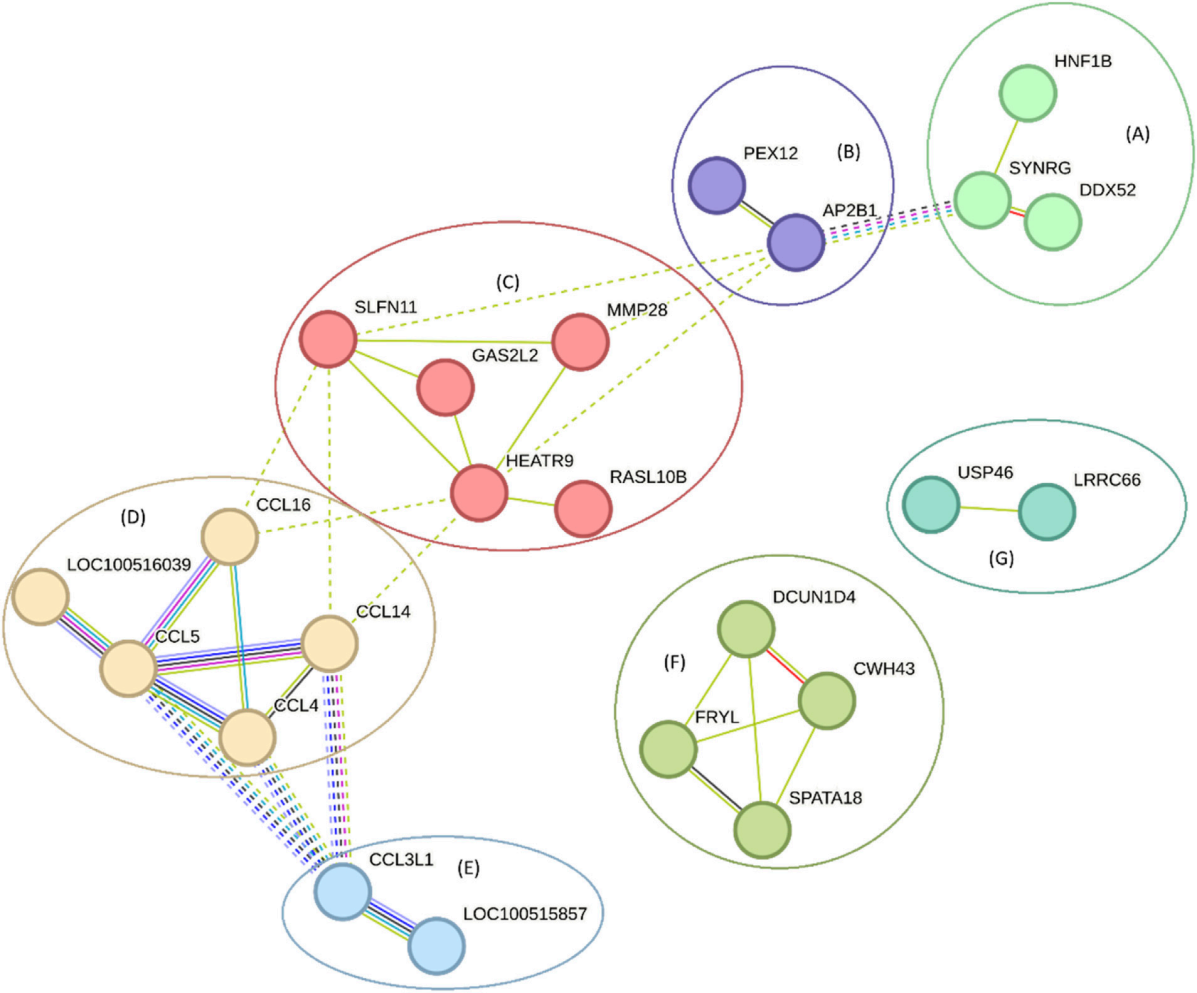
A network of genes annotated around eQTLs associated with blood biochemical parameters and cytokines from pig blood serum and liver tissue. The line thickness indicates the strength of data support, and the clusters are represented by letters (and colors). The clusters are **(A)** *SYNRG*, *DDX52*, and *HNF1B* **(B)** *AP2B1*, and *PEX12* **(C)** *HEATR9*, *RASL10B*, *GAS2L2*, *MMP28*, and *SLFN11* **(D)** *CCL14*, *CCL5*, *CCL4*, *CCL16*, and *LOC100516039* **(E)** *CCL3L1*, and *LOC100515857* **(F)** *CWH43*, *SPATA18*, *DCUN1D4*, and *FRYL* **(G)** *LRRC66*, and *USP46*. These results can also be accessed at https://version-12-0.string-db.org/cgi/network?networkId=bE85LK5REtmx.

A PPI network analysis for genes annotated around eQTLs associated with blood serum biochemical parameters and cytokines in *Sus scrofa*, which was performed to investigate their relationship with the annotated genes around eQTLs associated with biochemical parameters from pig blood serum and liver tissue are shown in Figure 10. The PPI network displayed an average node degree of 1.79 and a local clustering coefficient of 0.455, suggesting moderate connectivity among the nodes (medium confidence). The PPI enrichment p-value <10^−16^ indicates that the observed interactions are significantly more frequent than expected by chance, suggesting potential functional relationships among these genes. Clusters within the network were identified using Markov Cluster Algorithm (MCL) clustering with an inflation parameter of 3, highlighting groups of genes involved in processes such as chemotaxis and cytokine signaling pathways. The nodes without links were hidden, but the complete analysis can be accessed on https://version-12-0.string-db.org/cgi/network?networkId=bE85LK5REtmx and Supplementary Material 3.

## 4 Discussion

In this study, we used SNPs from medium-density SNP chip arrays (i.e., GGP-50K) and SNPs identified in the transcriptome of liver and skeletal muscle tissues to find cis- and trans-eQTL similar to the previous approach (Freitas et al., 2024). We subsequently evaluated their association with blood biochemical parameters, performance traits, and cytokine levels in skeletal muscle, liver, and blood of Large White pigs.

The number of eQTLs found, as shown in Figure 1, indicated the prevalence of cis-eQTLs compared to trans-eQTLs in skeletal muscle and liver tissues, which are in line with our previous findings (Freitas et al., 2024) and in the literature. For instance, Farhangi et al. (2024) identified 4,293 cis-eQTLs in liver tissue and 6,871 in muscle tissue, with cis-eQTLs showing stronger associations with their target genes compared to trans-eQTLs. By including SNPs detected in other regions of the genome, we enabled the detection of more distant associations (Freitas et al., 2024). However, liver tissue had a predominance of local regulation eQTLs, much greater when compared to skeletal muscle tissue. An important aspect of cis-eQTL detection is related to a greater ease (generally) of interpreting the effect of genetic modulation due to its proximity to the modulated gene. On the other hand, the greater the distance between the eQTL and the gene, the greater the complexity and difficulty of detection, as in the case of trans-eQTLs. However, the detection of both cis- and trans-eQTLs contributes to the understanding of genetic variability and regulatory mechanisms of important traits for animal production.

The Manhattan plots for IL-6 and IL-18 (Figure 4) in pig liver tissue highlight significant eQTL associations, particularly with the variant rs345667860 (3′UTR), which was identified using the VEP tool. This observation aligns with studies that focused on the regulatory influence of eQTL on cytokine expression in immune tissues, such as the work by Salnikova et al. (2020), who demonstrated the role of cytokine-related eQTLs in immune regulation. In the blood serum, the Manhattan plots for AST, HDL, and IL-6 also reveal significant eQTL associations, including the variants rs695637860 (downstream) and rs337362164 (missense). However, no significant associations were observed for the other traits. This may be due to the limited number of observations per trait (36 or 72), which reduced the power to detect associations between eQTLs and the studied traits. Furthermore, the use of only eQTL (1,199) for GWAS restricts the possibility of significant associations to a limited number of variants. The use of a larger number of SNPs could contemplate more associations with more traits. However, we considered only regulatory variants of gene expression for the GWAS analyses when selecting the eQTLs.

The enrichment of GO categories related to metabolic processes and protein binding (Figures 7, 8) is in line with the findings of previous studies that highlighted the role of metabolic pathways involving lipid metabolism in influencing feed efficiency and immune responses in pigs (Banerjee et al., 2020). In addition, Salnikova et al. (2020) identified enriched immune-related pathways, particularly those involving cytokine signaling, through eQTL analysis in the regulation of immune traits. In this sense indicating that eQTL associated with productive traits, biochemical parameters, and cytokines may be inserted into metabolic pathways related to the immune response, in the modulation of the physiological and productive characteristics of pigs. The representation of protein binding in the MF category resonates with the work of Kim et al. (2021), who identified key interactions between cytokines and their receptors, underlining the critical role of protein interactions in immune signaling pathways. Furthermore, the CC category is consistent with findings from studies on cytokine signaling, such as those by Salnikova et al. (2020), which demonstrated the involvement of membrane-associated proteins in immune regulation. This indicates a possible involvement of eQTLs in the immune response, since these proteins often serve as receptors or signaling molecules that mediate the immune response, further supporting the relevance of membrane-related components in the regulation of cytokine profiles and other immune-related traits.

The DAG and gene network illustrated in Figures 9, 10, focusing on eQTL associated with blood biochemical parameters and cytokines, highlights the interconnectedness of various genes involved in immune responses and metabolic processes. For instance, the cluster containing genes like *CCL5*, *CCL4*, and *CCL16* underscores the role of chemokines in mediating inflammatory responses, which is consistent with findings from studies that have shown the involvement of chemokine signaling pathways in immune regulation (Salnikova et al., 2020). These chemokines are known to be important in the recruitment of immune cells to sites of inflammation, suggesting an involvement in the immune system. Additionally, the involvement of genes such as *MMP28* and *HEATR9* within another cluster highlights the potential role of matrix metalloproteinases and heat shock proteins in tissue remodeling and stress responses. These findings are supported by research indicating that such genes play a role in both normal physiological processes and pathological conditions, particularly in the context of inflammation and immune response regulation (Banerjee et al., 2020). Moreover, clusters containing genes like *SYNRG* and *HNF1B* suggest potential involvement in regulatory networks that control cellular metabolism and gene expression, linking metabolic processes with immune function. This is in line with broader literature emphasizing the interconnected nature of metabolism and immunity, where metabolic pathways can influence immune cell function (Farhangi et al., 2024).

According to Fishbourne et al. (2013), chemokines such as *CCL2* and *CCL4* play a critical role in pigs infected with African swine fever virus (ASFV), particularly in cases involving high-virulence strains. The study demonstrated significant increases in *CXCL10* and *CCL2* expression, which correlates with our findings of significant eQTL associations with cytokines like IL-6 and IL-18. This suggests a direct link between the genetic regulatory mechanisms we identified, and the immune responses observed in pigs.

The protein encoded by the *CCL3L1* gene takes part in immune responses to viral infection (Fishbourne et al., 2013) and inflammation (Jaing et al., 2017; Kim et al., 2021), as evidenced by their upregulation in response to infection or activation of immune pathways (Kim et al., 2020). *CCL3L1* is also linked to cytokine-cytokine receptor interaction and natural killer cell-mediated cytotoxicity pathways (Jaing et al., 2017; Kim et al., 2021). The expression of these proteins is differentially regulated in response to various stimuli (Tada et al., 2020), indicating their interconnected roles in immune and inflammatory processes (Kim et al., 2021).

Our identification of rs695637860 as a local (cis-) eQTL modulating the *A0A286ZXF4* gene, which encodes a WAP domain-containing protein known for several functions, in this case as a serine endopeptidase inhibitor, and in the case of peptidases inhibition, protein digestion would be compromised. The association of this SNP with blood levels of AST and IL6 suggests a potential link between genetic variation and proteolytic enzyme activity, which could influence animal performance. The link between rs695637860 and levels of AST and IL-6 in the blood suggests that genetic variations in the *CCL3L1* region could influence liver function and systemic inflammation. Aminotransferases are enzymes that play a role in amino acid metabolism and are commonly used as biomarkers for liver health. Increased AST and IL-6 levels are indicative of an inflammatory response, which are associated with several inflammatory and autoimmune conditions. One might speculate is that the *CCL3L1* gene and its encoded protein *F1S1A1* may influence the expression of *A0A286ZXF4*, thereby modulating peptidase activity and consequently affecting inflammatory pathways. The upregulation of *F1S1A1* in response to infection or immune activation could lead to downstream effects on *A0A286ZXF4*, altering the balance of cytokine production and peptidase inhibition.

The identification of association between variants, such as rs345667860 and rs345667861 in liver tissue with IL-6 and IL-18, and rs695637860 and rs337362164 in serum with AST, HDL and IL-6, adds valuable insights into the genetic architecture underlying complex traits in pig production and health. However, as previously mentioned, there are possible limitations regarding the sample size, which could have reduced the statistical power to detect variants, especially those with a small effect. Future studies should aim to address these limitations by increasing sample sizes, expanding tissue types analyzed, and incorporating functional experiments to validate and further explore the roles of the identified genetic variants.

## 5 Conclusion

We identified associations between eQTL/SNPs and traits, including rs345667860 and rs345667861 associated with aspartate aminotransferase (AST) and interleukin-6 (IL-6) in liver tissue, and rs695637860 and rs337362164 associated with high-density lipoprotein (HDL) and IL-6 in blood serum. These eQTLs were significantly associated with the *A0A286ZXF4_PIG* (*WAP* domain-containing protein), *OCIAD2* (*OCIA* domain containing 2), and *TMCC3* (transmembrane and coiled-coil domain family 3) genes, which are all protein coding genes. These findings can also be applied in the development of genetic markers that may be used into strategies to predict the performance of animals in terms of health and production and contribute to understand how genetic variations relate to phenotypic traits in pigs.

## Data Availability

The datasets presented in this study can be found in online repositories. The names of the repository/repositories and accession number(s) can be found in the article/Supplementary Material.

## Glossary

ALB: Albumin (in g/dL)
AST: Aspartate aminotransferase (in U/L)
BFT: Backfat thickness measured by ultrasound in cm
BFT: Backfat thickness measured by ultrasound (in cm^2^)
BP: Biological Process
CC: Cellular Component
CCY: Cold carcass yield in percentage of the slaughter weight
CHOL: Cholesterol (in mg/dL)
DNA: Deoxyribonucleic acid
DP: Total coverage depth
eQTL: Expression Quantitative Trait Locus
eQTLs: Expression Quantitative Trait Loci
FDR: False Discovery Rate
GGP-50K: GeneSeek Genome Porcine medium density SNPs from SNP array
GLOB: Globulin (in g/dL)
GLU: Glucose (in mg/dL)
GO: Gene Ontology
GRM: Genomic Relationship Matrix between the pair of animals
GVCF: Genomic Variant Calling Format
HDL: High-density lipoprotein (in mg/dL)
HWE: Hardy-Weinberg Exact balance test
IFN-γ: Interferon-gamma (MFI)
IL-10: Interleukin-10 (in MFI)
IL-18: interleukin-18 (in MFI)
IL-1β: Interleukin-1 beta (in MFI)
IL-6: Interleukin-6 (in MFI)
IMF: Muscle fat content in percentage
Kb: Kilobase (1,000 base pairs)
LD: Linkage disequilibrium
LDL: Low-density lipoprotein (in mg/dL)
LEA: Loin eye area measured by ultrasound (in cm^2^)
LFC: Liver fat content (in %)
MAF: Minor allele frequency
Mb: Mega base pair
MF: Molecular function
MFI: median intensity fluorescence
MLMA: Mixed Linear Model Association
MP: Metabolic pathways
N: Number
PC: Principal components
QTL: Quantitative trait loci
QUAL: Phred score
**r** ^**2**^: Correlation
RNA: Ribonucleic acid
RNA-seq: RNA sequencing
SD: Phenotypic standard deviation
SNP: Single nucleotide polymorphism
SSC1: Sus scrofa chromosome 1
SSC18: Sus scrofa chromosome 18
SW: Slaughter weight (in kg)
SW: Slaughter weight in kg
TG: Triglycerides (in mg/dL)
TNF-α: Tumor necrosis factor-alpha (in MFI)
TP: Total proteins (in g/dL)
TPM: Transcripts per million
VCF: Variant calling format
VEP: Variant Effect Predictor
VLDL: Very low-density lipoprotein (in mg/dL)
