# Supplementary material for: Expression quantitative trait loci associated with performance traits, blood biochemical parameters, and cytokine profile in pigs
Source: Front Genet. 2025 Mar 5;16:1533424. doi: 10.3389/fgene.2025.1533424 (PMC11919875; doi:10.3389/fgene.2025.1533424)
Supplement: Supplementary file 1 [file DataSheet4.docx]

Supplementary Material

# Supplementary Data

**Supplementary Material 1.** Summary of eQTL. This file contains a comprehensive summary of eQTL (expression Quantitative Trait Loci) results, detailing associations between genetic loci and gene expression levels. The spreadsheet includes columns for SNP identifiers, genomic positions, gene names, and statistical metrics such as p-values and effect sizes, organized to facilitate the identification of cis and trans eQTLs. (.xlsx)

**Supplementary Material 2.** Manhattan and QQ plots of all traits (.zip). Supplementary Material 2. A directed acyclic graph (DAG) describing the hierarchical relationships between the enriched biological processes (.zip).

**Supplementary Material 3.** Support data to Figures 6, 7, 8, and 9.

## Supplementary Figures

**Supplementary Figure 1.** A complete DAG with all GO terms and metabolic pathways (.png). This image shows a Directed Acyclic Graph (DAG) with selected GO terms and metabolic pathways. Nodes represent GO terms or pathways, with edges indicating relationships, helping to visualize functional annotations.

## Supplementary Tables

The process of SNP data combination from the same animals

Supplementary Table 1. The process of SNP data combination from the same animals

| Merge steps | Initial SNPs count | New SNPs count | Step result |
| --- | --- | --- | --- |
| Liver and GGP-50k | 84,809 SNPs  71 liver samples  Genotype rate = 0.947 | 38,279 SNPs from GGP50k,  38,009 are new, while 270 are present in the liver. | 122,325 SNPs merged.  Genotype Rate 0.952. |
| Liver and GGP-50k SNPs merge with Muscle SNPs |  | 75,447 SNPs from muscle, of these, 24,019 are new, while 51,428 are present. | 146,344 SNPs  Muscle Liver and GGP50k  The genotyping rate is 0.925738. |

Supplementary Table 2. Step-by-step, in the order of Quality filters applied in the SNPs data set.

| Order^[[1]](#footnote-1)^ | Quality filters step | SNP count | Total |
| --- | --- | --- | --- |
| 1 | Initial SNPs number | 146,344 SNPs | 70,693 SNPs passed in the QC.  (genotype rate is 0.988686) |
| 2 | Missing genotype (> 95%) | 67,861 SNPs removed |  |
| 3 | Hardy-Weinberg exact test (< 1e-6) | 539 SNPs removed |  |
| 4 | Minor allele frequency (< 5%) | 7,251 SNPs removed |  |

Supplementary Table 3. The step-by-step of Linkage Disequilibrium (LD) pruning adopting a correlation between pair SNPs pair threshold (r2) threshold upper than 0.8.

| Step number | Pruning for LD (R2 > 0.8), 100kb window rolling by 1 variant per step |
| --- | --- |
| 1 | Pruned 2621 variants from chromosome 1, leaving 3,630 |
| 2 | Pruned 2037 variants from chromosome 2, leaving 3,039 |
| 3 | Pruned 1990 variants from chromosome 3, leaving 3,067 |
| 4 | Pruned 1797 variants from chromosome 4, leaving 2,324 |
| 5 | Pruned 1707 variants from chromosome 5, leaving 2,480 |
| 6 | Pruned 2283 variants from chromosome 6, leaving 3,139 |
| 7 | Pruned 1925 variants from chromosome 7, leaving 2,897 |
| 8 | Pruned 1607 variants from chromosome 8, leaving 2,258 |
| 9 | Pruned 2192 variants from chromosome 9, leaving 3,017 |
| 10 | Pruned 863 variants from chromosome 10, leaving 1,191 |
| 11 | Pruned 869 variants from chromosome 11, leaving 1,397 |
| 12 | Pruned 1813 variants from chromosome 12, leaving 2,067 |
| 13 | Pruned 1636 variants from chromosome 13, leaving 2,737 |
| 14 | Pruned 2041 variants from chromosome 14, leaving 3,028 |
| 15 | Pruned 1394 variants from chromosome 15, leaving 1,947 |
| 16 | Pruned 662 variants from chromosome 16, leaving 1,220 |
| 17 | Pruned 974 variants from chromosome 17, leaving 1,334 |
| 18 | Pruned 523 variants from chromosome 18, leaving 987 |
| Total variants removed | 28,934 |
|  | 41,759 SNPs from 72 animals. Total genotyping rate is 0.989. |

Supplementary Table 4. Descriptive statistics of tissues parameters from pigs

| TRAIT | N | Mean | Minimum | Maximum | SD |
| --- | --- | --- | --- | --- | --- |
| Albumin (g/dL) | 72 | 3.73 | 2.92 | 4.41 | 0.32 |
| Aspartate aminotransferase (U/L) | 72 | 44.44 | 27.00 | 171.00 | 21.16 |
| Backfat thickness (mm) | 72 | 14.68 | 9.90 | 23.10 | 2.53 |
| Cholesterol (mg/dL) | 72 | 96.73 | 70.70 | 132.10 | 12.03 |
| Cold carcass yield (%) | 72 | 69.94 | 66.36 | 72.97 | 1.23 |
| Globulin (g/dL) | 72 | 3.02 | 2.23 | 3.98 | 0.37 |
| Glucose (mg/dL) | 72 | 84.96 | 46.80 | 119.90 | 14.67 |
| High-density lipoprotein (mg/dL) | 72 | 43.32 | 29.90 | 61.10 | 6.56 |
| IFNg blood serum (MFI) | 72 | 21.14 | 7.00 | 129.00 | 18.53 |
| IFNg liver tissue (MFI) | 36 | 26.61 | 16.00 | 38.00 | 5.84 |
| IFNg muscle tissue (MFI) | 36 | 12.10 | 9.00 | 21.00 | 2.52 |
| IL-10 blood serum (MFI) | 72 | 69.40 | 15.00 | 528.00 | 100.94 |
| IL-10 liver tissue (MFI) | 36 | 296.85 | 125.00 | 716.00 | 131.01 |
| IL-10 muscle tissue (MFI) | 36 | 58.32 | 16.00 | 196.00 | 45.68 |
| IL-18 blood serum (MFI) | 72 | 68.00 | 22.00 | 479.00 | 66.81 |
| IL-18 liver tissue (MFI) | 36 | 951.14 | 337.00 | 1457.00 | 314.81 |
| IL-18 muscle tissue (MFI) | 36 | 108.06 | 30.00 | 1035.00 | 171.69 |
| IL-1b liver tissue (MFI) | 36 | 36.28 | 20.00 | 69.00 | 9.87 |
| IL-1beta blood serum (MFI) | 72 | 67.81 | 19.00 | 408.00 | 63.06 |
| IL-1β muscle tissue (MFI) | 36 | 18.06 | 13.00 | 39.00 | 5.39 |
| IL-6 blood serum (MFI) | 72 | 50.84 | 14.00 | 518.00 | 66.97 |
| IL-6 liver tissue (MFI) | 36 | 12.68 | 10.00 | 18.00 | 1.66 |
| IL-6 muscle tissue (MFI) | 36 | 9.25 | 8.00 | 13.00 | 1.00 |
| Intramuscular fat content (%) | 72 | 2.35 | 0.21 | 8.38 | 1.23 |
| Liver fat content (%) | 72 | 2.80 | 0.53 | 10.62 | 2.17 |
| Loin eye area (cm²) | 72 | 44.27 | 23.40 | 57.20 | 5.17 |
| Low-density lipoprotein (mg/dL) | 72 | 44.33 | 0.60 | 64.10 | 9.50 |
| Slaughter weight (Kg) | 72 | 132.71 | 107.00 | 160.00 | 10.93 |
| TNF-α blood serum (MFI) | 72 | 73.24 | 12.00 | 289.00 | 52.55 |
| TNF-α liver tissue (MFI) | 36 | 52.99 | 26.00 | 148.50 | 22.01 |
| TNF-α muscle tissue (MFI) | 36 | 17.71 | 11.00 | 110.00 | 16.20 |
| Total proteins (g/dL) | 72 | 6.75 | 5.70 | 7.79 | 0.46 |
| Triglycerides (mg/dL) | 72 | 45.40 | 17.50 | 265.80 | 29.71 |
| Very low-density lipoprotein (mg/dL) | 72 | 9.08 | 4.00 | 53.00 | 5.94 |

Albumin (ALB; in g/dL), aspartate aminotransferase (AST; in U/L), backfat thickness measured by ultrasound (BFT; in cm2), cholesterol (CHOL; in mg/dL), cold carcass yield as a percentage of the slaughter weight (CCY; in %), globulin (GLOB; in g/dL), glucose (GLU; in mg/dL), high-density lipoprotein (HDL; in mg/dL), intramuscular fat content (IMF, in %), liver fat content (LFC; in %), loin eye area measured by ultrasound (LEA; in cm2), low-density lipoprotein (LDL; in mg/dL), slaughter weight (SW; in kg), total proteins (TP; in g/dL), triglycerides (TG; in mg/dL), very low-density lipoprotein (VLDL; in mg/dL), and the cytokine levels in skeletal muscle, liver, and blood for Interleukin-10 (IL-10; in MFI), interferon-gamma (IFN-γ ; MFI), interleukin-1 beta (IL-1β; in MFI), interleukin-6 (IL-6; in MFI), interleukin-18 (IL-18; in MFI), tumor necrosis factor-alpha (TNF-α; in MFI); N: Number of records; SD: Phenotypic standard deviation.

**Supplementary Table 5.** Summary of genomic inflation factors of p-values resulting from integrated GWAS and eQTL analyses.

| TRAIT | N animals | N eQTLs | λ |
| --- | --- | --- | --- |
| Albumin (ALB; in g/dL) | 72 | 1199 | 0.902 |
| Aspartate aminotransferase (AST; in U/L) | 72 | 1199 | 0.876 |
| Backfat thickness measured by ultrasound (BFT; in cm2) | 72 | 1199 | 0.944 |
| Cholesterol (CHOL; in mg/dL) | 72 | 1199 | 0.989 |
| Cold carcass yield as a percentage of the slaughter weight (CCY; in %) | 72 | 1199 | 0.970 |
| Globulin (GLOB; in g/dL) | 72 | 1199 | 1.258 |
| Glucose (GLU; in mg/dL) | 72 | 1199 | 1.014 |
| High-density lipoprotein (HDL; in mg/dL) | 72 | 1199 | 1.252 |
| IFNg blood serum (MFI) | 72 | 1199 | 1.000 |
| IFNg liver tissue (MFI) | 36 | 1199 | 0.895 |
| IFNg muscle tissue (MFI) | 36 | 1199 | 0.971 |
| IL-10 blood serum (MFI) | 72 | 1199 | 1.037 |
| IL-10 liver tissue (MFI) | 36 | 1199 | 0.884 |
| IL-10 muscle tissue (MFI) | 36 | 1199 | 1.165 |
| IL-18 blood serum (MFI) | 72 | 1199 | 1.073 |
| IL-18 liver tissue (MFI) | 36 | 1199 | 1.016 |
| IL-18 muscle tissue (MFI) | 36 | 1199 | 1.115 |
| IL-1β blood serum (MFI) | 36 | 1199 | 1.158 |
| IL-1β liver tissue (MFI) | 72 | 1199 | 0.977 |
| IL-1β muscle tissue (MFI) | 36 | 1199 | 1.042 |
| IL-6 blood serum (MFI) | 72 | 1199 | 0.878 |
| IL-6 liver tissue (MFI) | 36 | 1199 | 1.048 |
| IL-6 muscle tissue (MFI) | 36 | 1199 | 1.071 |
| Liver fat content (LFC; in %) | 72 | 1199 | 1.031 |
| Liver fat content (LFC; in %) | 72 | 1199 | 0.944 |
| Loin eye area measured by ultrasound (LEA; in cm2) | 72 | 1199 | 1.013 |
| Low-density lipoprotein (LDL; in mg/dL) | 72 | 1199 | 1.041 |
| Slaughter weight (SW; in kg) | 72 | 1199 | 0.949 |
| TNF-α blood serum (MFI) | 72 | 1199 | 0.994 |
| TNF-α liver tissue (MFI) | 36 | 1199 | 0.855 |
| TNF-α muscle tissue (MFI) | 36 | 1199 | 1.082 |
| Total proteins (TP; in g/dL) | 72 | 1199 | 1.197 |
| Triglycerides (TG; in mg/dL) | 72 | 1199 | 1.068 |
| Very low-density lipoprotein (VLDL; in mg/dL) | 72 | 1199 | 1.178 |

λ = Genomic inflation factor, albumin (ALB; in g/dL), aspartate aminotransferase (AST; in U/L), backfat thickness measured by ultrasound (BFT; in cm2), cholesterol (CHOL; in mg/dL), cold carcass yield as a percentage of the slaughter weight (CCY; in %), globulin (GLOB; in g/dL), glucose (GLU; in mg/dL), high-density lipoprotein (HDL; in mg/dL), intramuscular fat content (IMF, in %), liver fat content (LFC; in %), loin eye area measured by ultrasound (LEA; in cm2), low-density lipoprotein (LDL; in mg/dL), slaughter weight (SW; in kg), total proteins (TP; in g/dL), triglycerides (TG; in mg/dL), very low-density lipoprotein (VLDL; in mg/dL), and the cytokine levels in skeletal muscle, liver, and blood for Interleukin-10 (IL-10; in MFI), interferon-gamma (IFN-γ ; MFI), interleukin-1 beta (IL-1β; in MFI), interleukin-6 (IL-6; in MFI), interleukin-18 (IL-18; in MFI), tumor necrosis factor-alpha (TNF-α; in MFI); N: Number of records; SD: Phenotypic standard deviation.

1. The order of quality filters applied can be affect in each quality filter applied. [↑](#footnote-ref-1)
