# Supplementary figures and images for: Expression quantitative trait loci associated with performance traits, blood biochemical parameters, and cytokine profile in pigs
Source: Front Genet. 2025 Mar 5;16:1533424. doi: 10.3389/fgene.2025.1533424 (PMC11919875; doi:10.3389/fgene.2025.1533424)

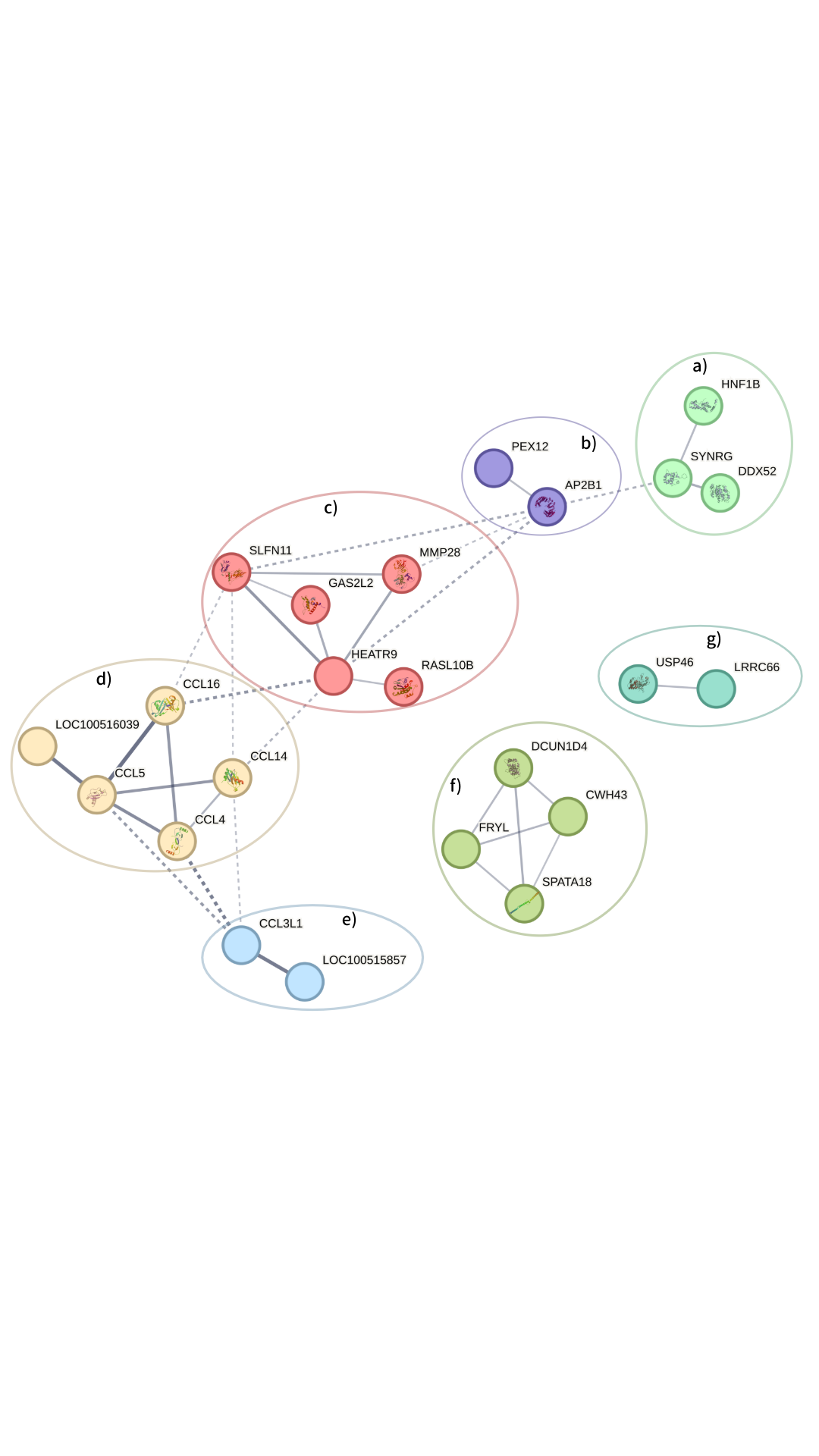

Supplement: Supplementary file 2 [file DataSheet3.zip › string_db_mediumconfidence_cluster_edited.png]

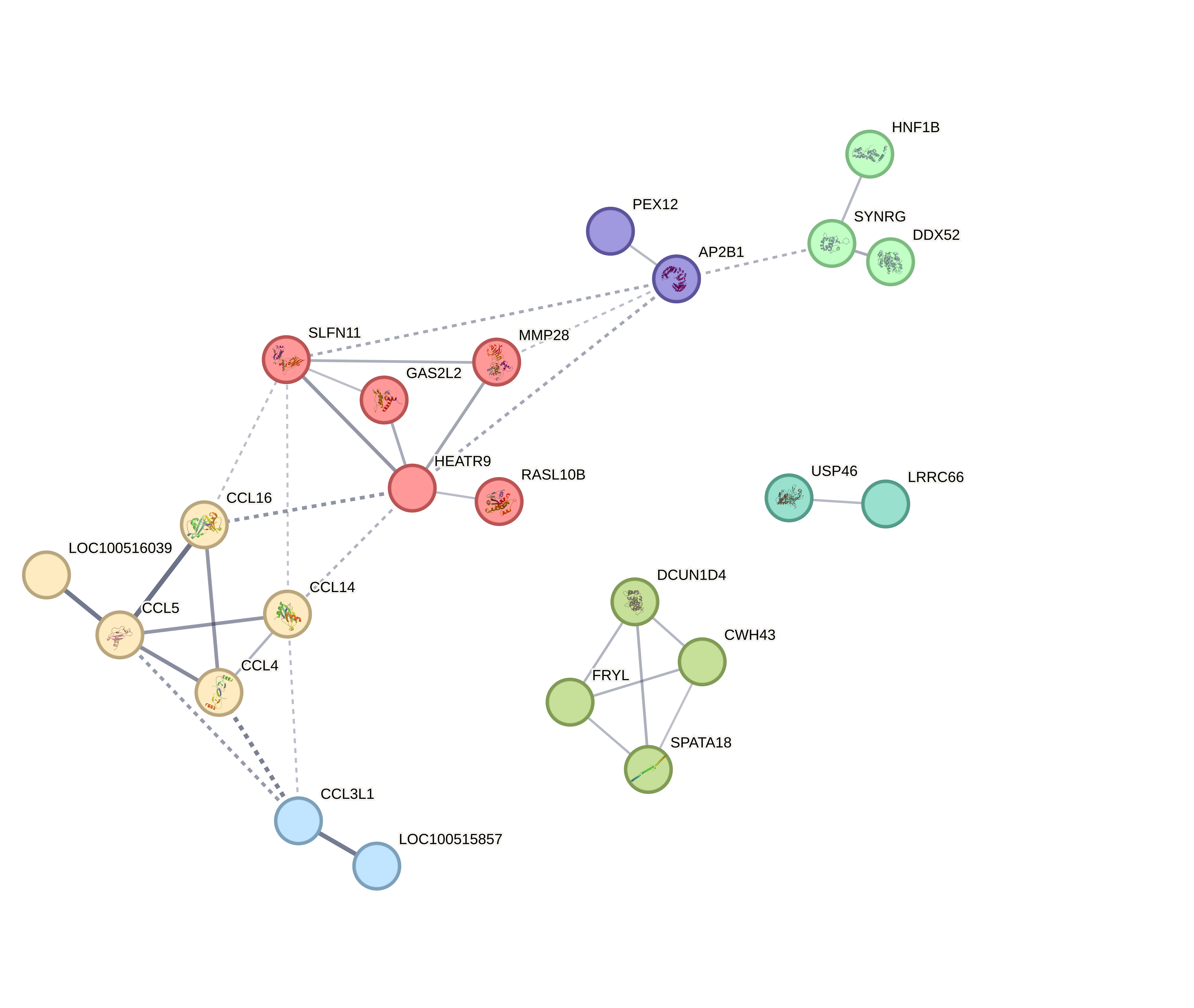

Supplement: Supplementary file 2 [file DataSheet3.zip › string_hires_image_confidence_medium_cluster.png]

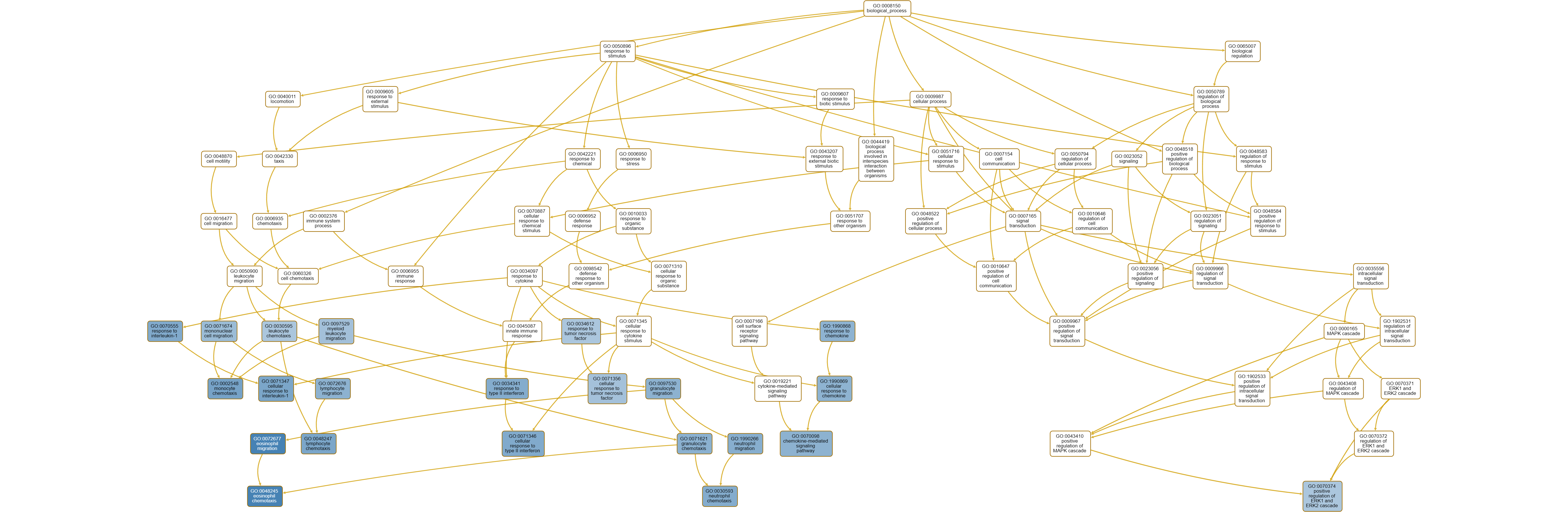

Supplement: Supplementary file 4 [file Image1.png]
